# Clear cell chondrosarcoma in Von Hippel-Lindau disease

**DOI:** 10.1007/s10689-019-00149-1

**Published:** 2019-10-31

**Authors:** Koen M. A. Dreijerink, Rachel S. van Leeuwaarde, Wenzel M. Hackeng, Rachel H. Giles, Wendy W. J. de Leng, Paul C. Jutte, Albert J. H. Suurmeijer, Bernadette P. M. van Nesselrooij, Lodewijk A. A. Brosens

**Affiliations:** 1 Department of Internal Medicine, Amsterdam University Medical Centers, Location VU University Medical Center, De Boelelaan 1117, 1081 HV Amsterdam, The Netherlands; 2 Department of Pathology, University Medical Center Utrecht, Utrecht, The Netherlands; 3 Department of Endocrine Oncology, University Medical Center Utrecht, Utrecht, The Netherlands; 4 Department of Nephrology and Hypertension, Regenerative Medicine Center, University Medical Center Utrecht, Utrecht, The Netherlands; 5 Department of Orthopedics, University Medical Center Groningen, Groningen, The Netherlands; 6 Department of Pathology, University Medical Center Groningen, Groningen, The Netherlands; 7 Department of Medical Genetics, University Medical Center Utrecht, Utrecht, The Netherlands

**Keywords:** Clear cell chondrosarcoma, Von Hippel-Lindau disease, *VHL* gene, Cyclin D1

## Abstract

A diagnosis of clear cell chondrosarcoma of the ulna was made in a patient with Von Hippel-Lindau disease (VHL). After surgery, genetic analysis of the tumor tissue showed loss of heterozygosity at the *VHL* gene locus. Immunohistochemical analysis confirmed loss of expression of the VHL protein in the tumor cells. In addition, abundant Cyclin D1 expression in the tumor was observed. Chondrosarcoma has been described before in a VHL patient and VHL protein expression has been correlated to tumor grade in a series of sporadic chondrosarcomas. In this report, we show that clear cell chondrosarcoma may be a rare but canonical VHL manifestation through a cell-autonomous mechanism involving somatic loss-of-heterozygosity of the *VHL* tumor suppressor gene. We discuss the relevance of this observation with regard to the pathogenesis of clear cell chondrosarcoma in the context of VHL.

## Introduction

Von Hippel-Lindau disease (VHL) is an autosomal dominantly inherited tumor syndrome that is caused by heterozygous inactivating mutations of the *VHL* tumor suppressor gene, usually in the germline [1]. Biallelic loss of *VHL* gene function through somatic loss of the second allele results in a state of pseudo hypoxia in cells that leads to angiogenesis and tumor or cyst formation [2]. VHL is characterized by tumors of the retina, brain and myelum (retinal and CNS hemangioblastomas), kidneys (clear cell renal cell carcinoma), adrenal glands (pheochromocytoma), endocrine pancreas (pancreatic neuroendocrine tumors) and endolymphatic sac and epididymis and broad ligament cystadenomas, in addition to potentially clinically relevant complex and simple cysts in the kidney, pancreas, adrenal gland, and associated with CNS hemangioblastomas (syrinx).

Chondrosarcomas are malignant tumors of the bone with variable morphology and clinical behavior that are characterized by the production of cartilage matrix. We report a case of clear cell chondrosarcoma in a VHL patient. Genetic analysis showed loss of heterozygosity (LOH) at the *VHL* gene locus. Loss of expression of the VHL protein (pVHL) suggests that clear cell chondrosarcoma may be part of the VHL tumor spectrum.

## Subject and methods

**Fig. 1:**
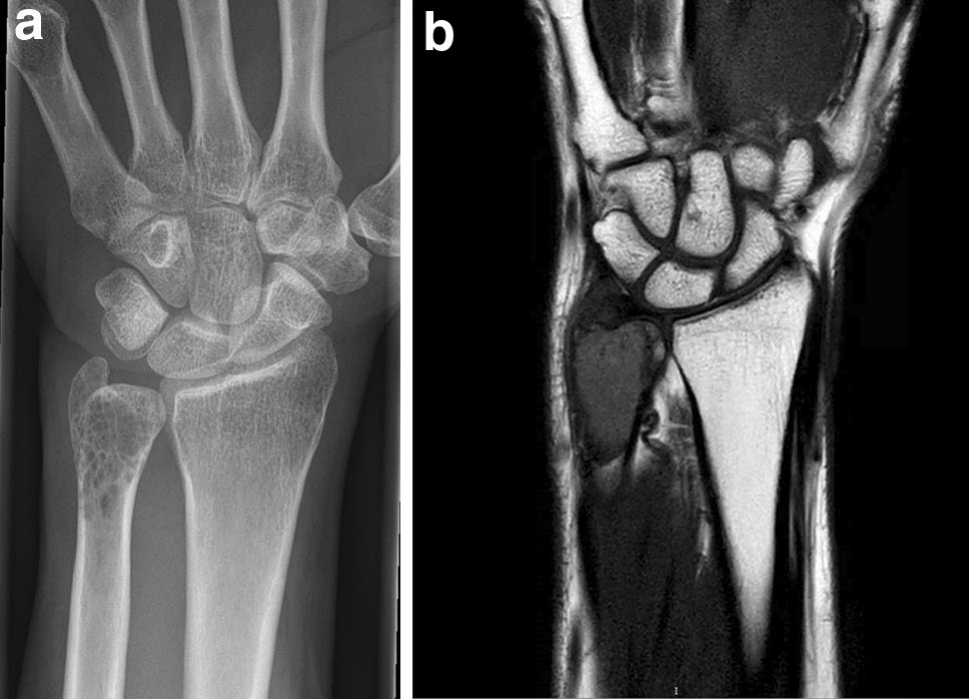
Imaging of a clear cell chondrosarcoma in a VHL patient. **a** X-ray of the left forearm. The lesion is located in the distal ulna. **b** Magnetic resonance imaging of the lesion

At age 48, a female VHL patient presented with pain in her left arm. She was diagnosed with VHL at age 25. Genetic testing of peripheral blood had confirmed a c.500G > A (p.Arg167Gln) *VHL* gene mutation. Her medical history included surgery for central nervous system hemangioblastomas at age 26, 28, 39 and 45 as well as a retinal hemangioma for which she underwent a coagulation procedure at age 26. Imaging studies suggested a lytic lesion in the left distal ulna (Fig. 1). A biopsy was taken that was suggestive of clear cell chondrosarcoma. Additional immunohistochemical analysis showed strong S-100 staining. Antibodies directed at cytokeratins 8, 18, AE1-3 and also inhibin, which are used for the identification of renal cell carcinoma or hemangioblastoma, were negative. Bone scintigraphy did not reveal any other bone lesions. Surgical resection resulted in complete removal of the tumor. Tumor tissue was frozen after surgery. DNA was isolated and after PCR amplification the *VHL* gene locus was analyzed by Sanger sequencing [3]. In addition, tumor DNA was subjected to comprehensive genetic and epigenetic analysis using an Ion Ampliseq Cancer Hotspot v2 panel (Thermo Fisher Scientific), Infinium CytoSNP-850 K and Infinium MethylationEPIC-850 K beadchip (Illumina). In parallel, tissue was processed for immunohistochemical analysis using monoclonal antibodies directed at pVHL and the pVHL-suppressed target Cyclin D1 (VHL, Cat.no. 556347, 1:200, BD Biosciences; Cyclin D1(SP4), Cat.no. 241R-15, 1:100, Cell Marque). The patient was informed and gave written permission for this case report.

## Results

**Fig. 2:**
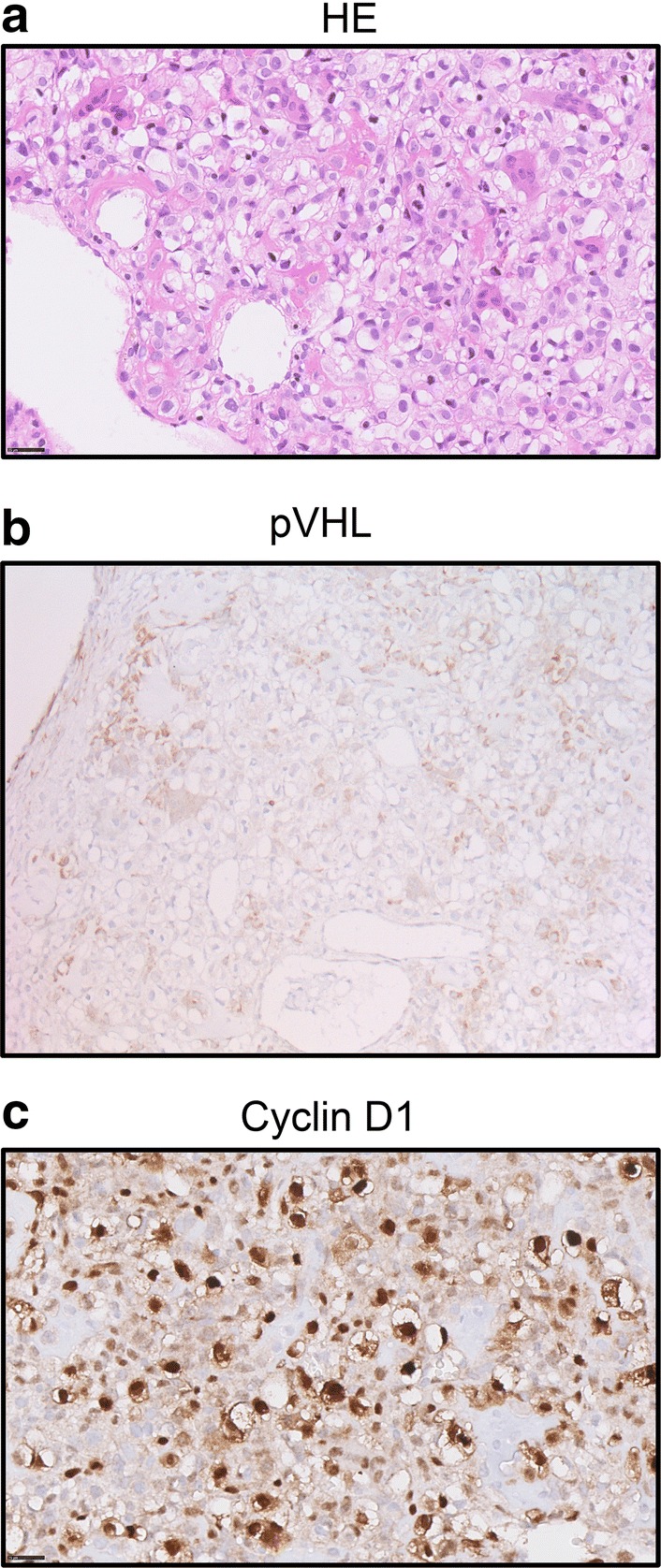
Molecular analysis of the tumor after surgical removal of the clear cell chondrosarcoma. **a** Hematoxylin and eosin (HE) stain of the tumor tissue (× 40 magnification). The tumor is composed of cells with distinct borders and clear to slightly eosinophilic cytoplasm with central nuclei. Osteoclast-type giant cells, reactive bone and wide vessels are present in the tumor. **b** Immunohistochemical staining of the tumor tissue using antibodies directed at pVHL (20 ×). Positive staining is observed in non-neoplastic multinucleated giant cells and other histiocytic cells present in the tumor, which are CD68 positive (not shown). **c** Immunohistochemical staining using antibodies directed at Cyclin D1 shows abundant expression in the tumor cells (×40)

**Fig. 3:**
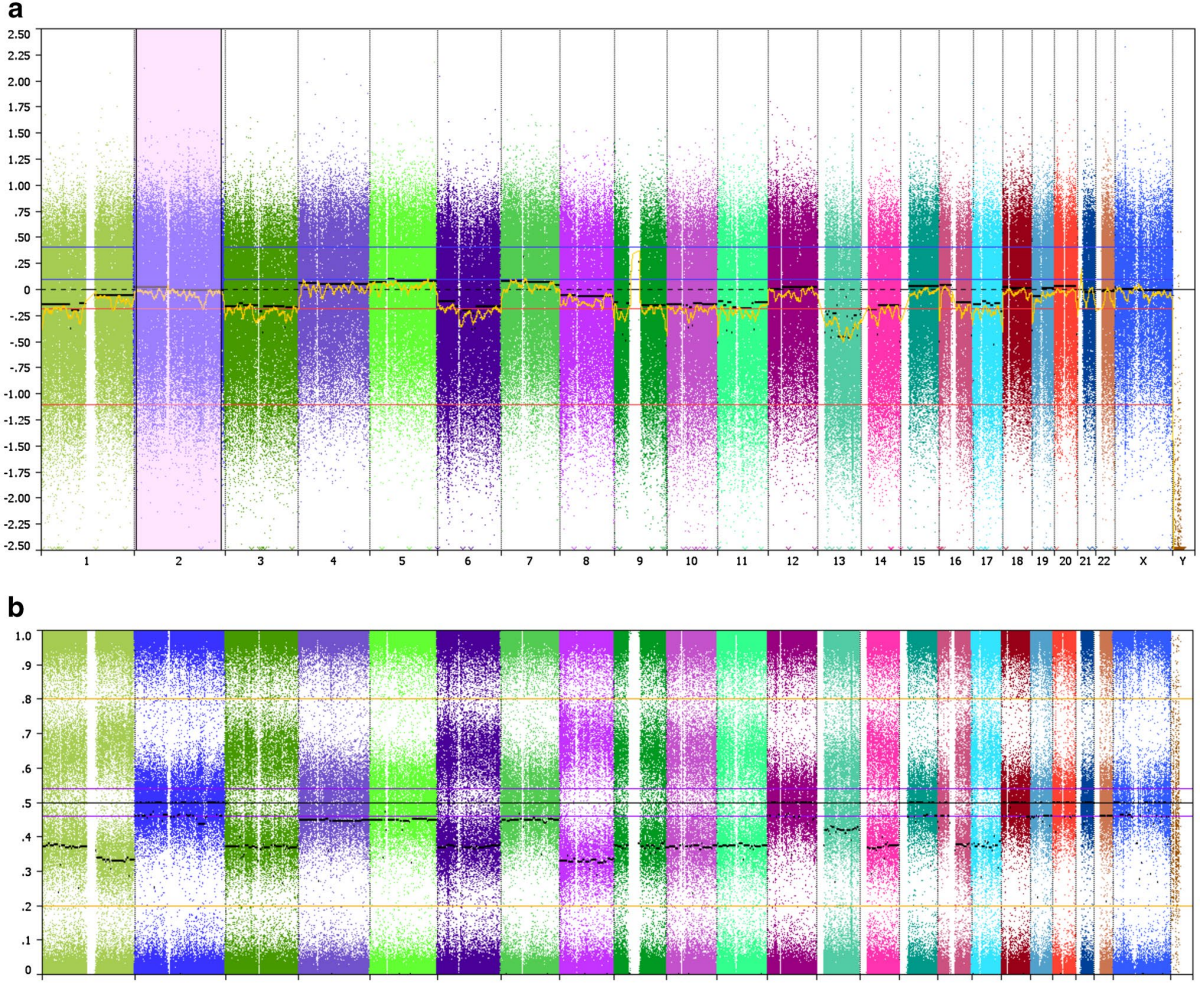
SNP array indicating chromosomal gains and losses in the chondrosarcoma. **a** Copy number variations for each chromosome (log2 ratio). **b** B-allele frequencies

Histological examination showed a tumor composed of sheets of cells with clear to slightly eosinophilic vacuolated cytoplasm and central nuclei with small central nucleoli (Fig. 2a). Numerous osteoclast-type giant cells and reactive bone cells were present in between the tumor cells. The estimated tumor cell fraction of the micro dissected material was 80%. Genetic analysis of the micro dissected tissue showed a higher chromatographic signal of the mutant allele than the wild type allele, suggestive for loss of the wild type allele (data not shown). Gene panel analysis confirmed the *VHL* gene mutation in the tumor. No other mutations were found, except a non-pathogenic variant of the ataxia-telangiectasia mutated (*ATM*) gene (c.1810C > T; p.(Pro604Ser)). Allelic loss of *VHL* was confirmed by SNP array (Fig. 3). Copy number variations included a monosomal pattern of chromosomes 3 (harboring the *VHL* gene), 6, 9 (*CDKN2A*), 10, 11, 13, 14, and 17. Also loss of 1p and 16q was found and copy neutral LOH at 1q and 8. The methylation array confirmed the above described copy number profile. No aberrant CpG methylation was observed at the *VHL* promoter (data not shown). To determine the effect of the loss of the wild type *VHL* allele on pVHL expression, we subsequently performed immunohistochemistry. Distinctly lower levels of pVHL were observed in the tumor cells as compared to adjacent non-neoplastic cells (Fig. 2b). These results indicate that a second genetic hit occurred in *trans* during tumor development that resulted in loss of pVHL. Profound expression of Cyclin D1 was observed in the tumor (Fig. 2c).

## Discussion

We describe a case of a VHL patient with a primary clear cell chondrosarcoma. Loss of function of the wild type *VHL* allele in the tumor and loss of pVHL expression suggest that the chondrosarcoma is a canonical VHL manifestation in this patient. Cyclin D1 could be the driver of cell proliferation in this tumor.

Approximately 75–90% of chondrosarcomas are so-called conventional chondrosarcomas (reviewed in [3]). These tumors rarely present in childhood or adolescence and most often are located in the tubular bones [4]. In conventional chondrosarcomas, mutations in the isocitrate dehydrogenase (*IDH*) genes are the most prevalent genetic events, in up to 59% of cases in a comprehensive exome sequencing study [5]. In addition, *TP53* gene function (20%), the retinoblastoma pathway (33%) and hedgehog signaling (18%) are affected by somatic mutations in the tumors. Previously, a mildly differentiated chondrosarcoma has been reported in a VHL patient. However, no genetic or immunohistochemical analysis of the tumor was performed in this patient [6]. Chen et al. assessed *VHL* mRNA and protein levels in a series of conventional chondrosarcomas [7]. Levels of both *VHL* mRNA and protein were found to be reduced in a significant proportion of chondrosarcomas compared with adjacent normal tissue. Low expression of pVHL was associated with apoptosis and tumor grade.

Clear cell chondrosarcoma is a rare subtype of non-conventional chondrosarcoma comprising approximately 2% of all chondrosarcoma cases and is considered a low-grade variant. It occurs predominantly at the epiphyseal ends of long bones. There are no known inherited causes for clear cell chondrosarcoma. Due to its rarity, limited information is available on the molecular background of this subtype. Meijer et al. reported that expression of the protein p16Ink4a, encoded by the *CDKN2A* gene, was absent in 20 out of 21 sporadic clear cell chondrosarcoma cases [8]. P16 inhibits expression of the Cyclin D1 (*CCND1*) gene and high expression of *CCND1* was observed in 42% of tumors tested. We also found LOH at the *CDKN2A* gene locus and abundant Cyclin D1 expression in the tumor in this VHL patient. In collaboration with cyclin-dependent kinases (CDK) 4 and 6, Cyclin D1 stimulates progression through the G1 phase of the cell cycle via retinoblastoma protein inactivation, which may lead to tumor formation. CDK4/6 inhibitors represent a promising new cancer treatment option.

The clear cell histology in renal cell carcinoma is known to be a result of accumulation of cytoplasmic lipids and glycogen. Lipid accumulation has been described in other VHL-associated tumors, such as pancreatic neuroendocrine tumors [9]. Although speculative, it is interesting to consider that the role of pVHL in clear cell chondrosarcoma is analogous and initiates similar metabolic changes in tumor development. The VHL protein functions as part of a ubiquitin E3 ligase that targets the alpha-subunits of the hypoxia inducible factor (HIF) by inducing oxygen-dependent degradation through ubiquitylation. Accumulation of nuclear HIF in a cell that has lost pVHL function results in permanent transcription of hypoxia-induced genes [2]. Interestingly, the *CCND1* gene, which has been found up regulated in clear cell chondrosarcomas, has also been reported as a pVHL-dependent HIF target gene in renal cell carcinoma [10]. Thus, the strong Cyclin D1 expression observed in the VHL tumor may be a direct result of the loss of pVHL-dependent transcriptional repression. Due to their pseudo hypoxic state, VHL-related tumors tend to be highly vascularized and anti-angiogenic compounds are routinely used for the treatment of advanced disease. Chondrosarcomas have also been successfully treated with anti-angiogenic drugs, further indicating common underlying mechanisms of development and treatment of these tumor types.

To conclude, we report a clear cell chondrosarcoma in a VHL patient that exhibited loss of pVHL expression due to loss of expression of the *VHL* gene. Our data support the notion that clear cell chondrosarcoma could be a rare but specific manifestation of VHL. Increased Cyclin D1 function leading to vascularized clear cell tumors may be a common molecular feature of VHL-associated tumors and clear cell chondrosarcoma, which could be useful for therapeutic purposes in the future.
